# Do Cerebral Small Vessel Disease and Multiple Sclerosis Share Common Mechanisms of White Matter Injury? A Genetic Study

**DOI:** 10.1161/STROKEAHA.118.023649

**Published:** 2019-06-21

**Authors:** Robin B. Brown, Matthew Traylor, Stephen Burgess, Stephen Sawcer, Hugh S. Markus

**Affiliations:** 1 From the Department of Clinical Neurosciences (R.B.B., M.T., S.S., H.S.M.), University of Cambridge, United Kingdom; 2 Cardiovascular Epidemiology Unit, Department of Public Health and Primary Care (S.B.), University of Cambridge, United Kingdom; 3 MRC Biostatistics Unit, Cambridge Institute of Public Health, United Kingdom (S.B.).

**Keywords:** genetic association studies, inflammation, magnetic resonance imaging, multiple sclerosis, white matter

## Abstract

**Background and Purpose—:**

The role of inflammation in ischemic white matter disease is increasingly recognized, and further understanding of the pathophysiology might inform future treatment strategies. Multiple sclerosis (MS) is a chronic autoimmune condition in which inflammation plays a central role that also affects the white matter. We hypothesized that white matter injury might share common mechanisms and used statistical genetics techniques to assess whether having genetically elevated white matter hyperintensity (WMH) volume was associated with increased MS risk.

**Methods—:**

We investigated the genetic association in 2 cohorts with magnetic resonance imaging-quantified ischemic white matter lesion volume (WMH in stroke; n=2797 and UK Biobank; n=8353) and 14 802 cases of MS and 26 703 controls from the International Multiple Sclerosis Genetics Consortium. We further performed individual-level polygenic risk score calculations for MS and measures of structural white matter disease in UK Biobank. Finally, we looked for evidence of overlapping risk across the whole genome.

**Results—:**

There was no association of genetic variants influencing MS with WMH volume using summary statistics in the WMH in stroke cohort (relative risk score =1.014; 95% CI, 0.936–1.110) or in the UK Biobank cohort (relative risk score =1.030; 95% CI, 0.932–1.117). Conversely, assessing the contribution of single nucleotide polymorphisms significantly associated with WMH on the risk of MS there was no significant association (relative risk score =0.930; 95% CI, 0.736–1.191). There were no significant associations between polygenic risk scores calculations; these results were robust to the selection of single nucleotide polymorphisms at a range of significance thresholds. Whole genome analysis did not reveal any overlap of risk between the traits.

**Conclusions—:**

Our results do not provide evidence to suggest a shared mechanism of white matter damage in ischemia and MS. We propose that inflammation acts in distinct pathways because of the differing nature of the primary insult.

## Abstract 2

Supplemental Digital Content is available in the text.

Cerebral small vessel disease (SVD) causes a quarter of all strokes in the form of lacunar infarcts and is the most common pathology underlying vascular cognitive impairment. White matter hyperintensities (WMHs), best seen on T2-weighted magnetic resonance imaging (MRI), are an important radiological feature of SVD and their presence predicts both stroke and dementia.^1^ Despite their importance, understanding of their underlying pathophysiology is incomplete, and there are few effective treatments.

Recently, a role of inflammation in the genesis and progression of WMH in SVD has been proposed,^2^ with many lines of evidence supporting this hypothesis. Postmortem samples show inflammatory cells in the white matter around blood vessels and in the vicinity of demyelination.^3^ Proinflammatory enzymes are elevated in the cerebrospinal fluid of patients with vascular cognitive impairment^4^ and blood biomarkers of inflammation, including IL (interleukin)-6 and CRP (C-reactive protein), have been associated with the presence of WMHs.^5^ Plasma levels of inflammatory cytokines, markers of oxidative stress (such as myeloperoxidase), and vascular inflammation (such as matrix metalloproteases) have been associated with the WMH volume,^6–8^ while there is evidence that ICAM-1 (intercellular adhesion molecule 1) levels in serum are elevated^9^ and predict WMH progression in longitudinal studies.^10^ Furthermore, in animal models of SVD, glia in the white matter are activated preferentially in response to ischemia^11^ and matrix metalloproteases and tumour necrosis factor-α colocalize within areas of white matter disease.^12^ Whether these inflammatory processes occur in disease pathogenesis itself or in response to white matter injury remains unclear.

In contrast, the immune system plays a central role in the pathogenesis of multiple sclerosis (MS). Association with variation in the major histocompatibility complex is well recognized,^13^ and genome-wide association studies (GWAS) have revealed many additional single nucleotide polymorphisms (SNPs) that also influence the risk of developing the disease, the majority of which map close to immunologically relevant genes.^14,15^

In view of the fact that both MS and SVD result in white matter high signal lesions on MRI, and share a number of pathological inflammatory features, we hypothesized that shared inflammatory pathways might mediate the response to neuronal injury in both conditions. This might provide further insight into the pathological mechanisms of these diseases and offer novel targets for future treatments in SVD. Indeed, early work in a rat model has shown that dimethyl fumarate, a treatment for relapsing-remitting MS, reduces the level of glial activation and the deterioration in specific measures of neuronal function after hypoperfusion.^16^

Increasingly, genetics is being used to determine whether potentially overlapping conditions share common disease mechanisms, and this approach has, for example, been successfully used to demonstrate sharing of genetic risk between SVD and Alzheimer disease.^17^ To investigate whether ischemic WMH and MS might share common mechanisms leading to white matter damage, we used GWAS data to determine whether having genetically elevated WMH levels was associated with increased MS risk, or the reverse, whether having genetically elevated MS risk was associated with increased WMH levels.

## Methods

Published summary statistics are available in the online-only Data Supplement to the referenced GWAS studies. MS meta-analysis data are available from the International Multiple Sclerosis Genetics Consortium. Further data that support the findings from this study are available from the corresponding author at a reasonable request.

All original individual studies received approval from respective local ethics committees; the participants for each study gave informed written consent.

### Study Populations

#### Ischemic White Matter Damage—WMHs

Risk scores for ischemic white matter disease were calculated in 2 independent cohorts.

1. In a cohort of patients with ischemic stroke (WMH in stroke; n=2797). This population has been described previously^18^ but in brief comprises several independent cohorts of patients with acute ischemic stroke enrolled through hospital-based studies from 1995 to 2013. Data were contributed from the Welcome Trust Case Control Consortium 2 study which included patients from Edinburgh, Oxford, St George’s (University of London) and Munich and from the Milano, GENESIS (Identifying Genetic Risk Factors for Cerebral Small Vessel Disease and Leukoaraiosis; 1–3), Leuven, SLESS (South London Ethnicity and Stroke Study) and UK DNA Lacunar studies. Patients with a proven or suspected monogenic cause of stroke or with other nonischemic diseases affecting white matter were excluded.
2. In a prospective population-based cohort (UK Biobank; n=8353). UK Biobank is a prospective study that recruited over 500 000 participants aged 40 to 69 from 2006 to 2010. (http://www.ukbiobank.ac.uk). Participants are followed-up by health records, questionnaires, physiological measurements, blood tests, and imaging. We used the second release of the MRI data from the subset of patients who underwent brain MRI (n=9066). Patients with a diagnosis of stroke (self-reported or based on health records), MS, or other neurodegenerative disease were excluded.

#### Multiple Sclerosis

Summary statistics were provided by the International Multiple Sclerosis Genetics Consortium and are based on their latest meta-analysis,^19^ which included 14 802 cases meeting internationally agreed clinical and paraclinical criteria^20^ and 26 703 healthy controls.

### WMH Lesion Volume Estimation

In the WMH in stroke population, WMHs were quantified on T2-fluid attenuated inversion recovery (when available) or T2 MRI, using the contralateral hemisphere to the stroke as described previously^18^ by trained and blinded raters. The GENESIS 3 and SLESS data sets were analyzed using Jim image analysis software version 7.0.5 (Xinapse Systems Limited, http://www.xinapse.com/j-im-7-software/), a semi-automated program in which a region of interest containing voxels above a particular threshold value is defined and then manually adjusted. The remaining subsets were analyzed using DISPunc,^21^ a semi-automated program where a starting voxel (seed) was marked by the rater and then outlined automatically following the direction of the maximum signal intensity gradient at each voxel to delineate the lesion. WMH volumes were doubled to provide an estimate for the whole brain and corrected for total intracranial volume and log-normalized.

In the UK Biobank population, WMHs were quantified on T2-fluid attenuated inversion recovery (when available) or T2 MRI using the Brain Intensity Abnormality Classification Algorithm.^22^ This is a semi-automated method for WMH detection based on the k-nearest neighbor algorithm; the total WMH volume was calculated from the white matter voxels exceeding the probability of 0.9 of being WMH, corrected for total intracranial volume and log-normalized. The analysis was performed on the WMH volume imaging derived phenotype available from UK Biobank.^23^

Diffusion tensor imaging parameters, such as fractional anisotropy (FA) and mean diffusivity (MD), have been shown to be more sensitive measures of disruption of white matter microarchitecture than WMH on T2-fluid attenuated inversion recovery^24^; for this reason, we also assessed the contribution of these SNPs to FA and MT measures in the UK Biobank population. We used the imaging derived phenotypes provided by UK Biobank.^23^ Corrected images were projected on to standardized templates comprising 48 neuronal tracts to produce mean FA and MD values for each tract. Principal component analysis was then used to extract the first principal component for FA and MD; these were taken forward as latent variables for further analysis. This method has been previously been used to analyze diffusion tensor imaging in participants in UK Biobank.^25^

### Selection of Genome-Wide Significant SNPs

We combined the lists of SNPs associated with MS at genome-wide significance in previous GWAS.^14,15^ From this list of variants, we excluded SNPs which did not reach significance at *P*<5×10^−8^ and pruned by linkage equilibrium^26^ (*r*^2^>0.1) to define a list of 106 variants to take into further analysis. This list of SNPs is given in the online-only Data Supplement (Table I in the online-only Data Supplement).

We further defined a set of 11 SNPs that are significantly associated (*P*<5×10^−8^) with WMH volume in existing GWAS studies^18,27,28^ and in linkage equilibrium. This list is provided in the online-only Data Supplement (Table II in the online-only Data Supplement).

### Statistical Analysis

#### Summary-Level Genetic Risk Score Analysis

We first assessed the effect of the MS SNPs on WMH volumes using the inverse variance weighted summary statistic method described by Ehret et al,^29^ calculating a weighted multi-SNP risk score for the cumulative effect of these SNPs on WMH lesion volume. This is similar to conventional Mendelian randomization analysis but relies on rather less strict assumptions about one condition directly causing another.^30^ If the 2 conditions have shared underlying mechanisms, then we would expect genetic predictors of MS risk to be associated with WMH volume, and for the genetic association with the inverse variance weighted risk score (which is a weighted average of the genetic associations with the SNPs in the score) to differ from zero (and vice versa). We then performed the same analysis assessing the influence of MS SNPs on MD and FA from genome-wide analyses in UK Biobank.^25^ All analysis was performed in the R Project for Statistical Computing^31^ using the gtx package.^32^ This is a toolbox that regresses the risk scores of SNPs for a response trait on to the calculated weighted multi-SNP risk scores of a dependent trait.

#### Individual-Level Genetic Risk Score Analysis

Second, we tested the contribution of the set of MS SNPs to ischemic white matter disease by calculating polygenic risk scores across these SNPs for each subject in the UK Biobank data set.^33^ We assessed whether the MS risk score contributed to WMH volume, FA, or MD in a linear regression model that included genotyping batch, age, sex, and the first 10 ancestry informative principal components as covariates. For this analysis, we used 4 sets of SNPs to enable a comprehensive assessment of potential shared mechanisms. We first included only SNPs reaching genome-wide significance with MS, as described above. We then included SNPs reaching 3 thresholds: *P*<1×10^−4^, *P*<0.05, and *P*<0.5. For these SNPs, we performed linkage disequilibrium (LD)-based clumping to derive a set of independent SNPs with either *r*^2^<0.01or 1000 Mb between all pairs of SNPs.

#### LDSCORE Analysis

Finally, we assessed the global genetic correlation between MS and WMH, FA, and MD, using the LD score regression approach^34^ based on summary statistics. Briefly, this approach assumes that LD blocks containing higher numbers of significantly correlated SNPs are more likely to represent significant causal regions and that this information can be used to determine heritability and coheritability of pairs of traits. LD scores are, therefore, calculated as the sum of *r*^2^ values within an LD block and regressed against the product of SNP *Z* statistics in given LD blocks for pairs of traits to obtain an estimate of genetic correlation. This analysis used the ldsc package (https://github.com/bulik/ldsc) based on precomputed LD scores from European populations (https://data.broadinstitute.org/alkesgroup/LDSCORE/eur_w_ld_chr.tar.bz2).

## Results

We found no significant association of genome-wide significant variants influencing MS with WMH volume using summary statistics in the WMH in stroke cohort (relative risk score =1.014; 95% CI, 0.936–1.100) or in the UK Biobank cohort (relative risk score =1.030; 95% CI, 0.932–1.117). Similarly, there was no significant association of genetic variants influencing MS with either FA (relative risk score =0.991; 95% CI, 0.878–1.119) or MD (relative risk score =0.972; 95% CI, 0.863–1.094; Table 1).

**Table 1.**
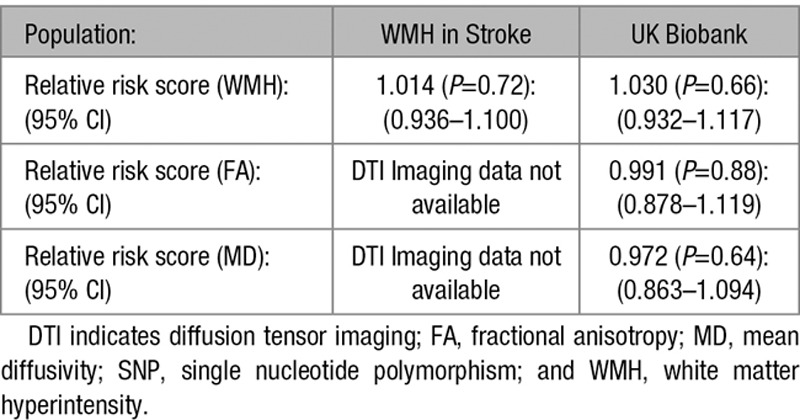
Relative Multi-SNP Genetic Risk Scores for WMH Lesion Volume, FA, and MD Across 2 Independent Populations

We next performed the converse analysis, assessing the contribution of SNPs significantly associated with WMH on the risk of MS. Again, we found no significant association (relative risk score =0.930; 95% CI, 0.736–1.191). Sensitivity analysis of all association test results was performed by repeating these measures using minor allele frequency thresholds of 10% and 20%; these results were also null (Table III in the online-only Data Supplement).

Third, we performed genetic risk score analysis based on individual-level data from UK Biobank for 4 *P* value thresholds: *P*<5×10^−8^, *P*<1×10^−4^, *P*<0.05, and *P*<0.5. We found no statistically significant association between this MS risk score and WMH volume, FA, or MD at any *P* value threshold (Table 2).

**Table 2.**
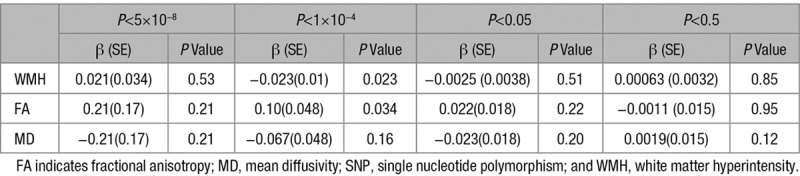
Association of Polygenic Risk Scores Derived From Multiple Sclerosis Associated SNPs at Given P Value Thresholds With WMH, FA, and MD in UK Biobank

Finally, we estimated the genome-wide genetic correlation between MS and WMH volume, FA, and MD from UK Biobank, using LDSCORE regression. Again, there was no evidence of shared genetic effects between MS and WMH volume (rG =0.037; SE=0.088; *P*=0.68), FA (rG =0.083; SE=0.077; *P*=0.28), or MD (rG =0.097; SE=0.095; *P*=0.31), respectively.

## Discussion

We sought to determine, based on sharing of genetic susceptibility factors, whether shared pathways underlie both ischemic white matter damage and MS. We found no evidence that SNPs that are significantly associated with MS affect the risk of WMH volume nor that SNPs that are significantly associated with WMH affect the risk of MS. This was true for both SNPs that are established as being associated with both diseases using genetic risk score approaches, and for genome-wide SNPs at lower significance thresholds using LDSCORE and polygenic risk score approaches. No SNP associated significantly with both condition at the genome-wide significance level (*P*<5×10^−8^).

As diffusion tensor imaging measures have been shown to be a more sensitive measure of ischemic white matter injury and found to correlate better with clinical and cognitive parameters than WMH volume,^35,36^ we also looked for sharing of genetic risk between diffusion tensor imaging measures of white matter damage and MS. Again, we found no evidence that genetic risk was shared.

Inflammation is a primary disease mechanism in MS, and inflammatory processes have also been implicated in ischemic white matter damage. However, determining whether inflammation plays a causal role in disease pathogenesis in ischemic WMH is challenging. Much of the evidence, for example, from pathological and observational data, show an association but does not exclude the possibility that the inflammation occurs secondary to tissue damage. As genomes are randomly allocated at conception stronger claims about causality can be made based on genetic associations, so long as certain assumptions are not invalidated.^37^ Using genetic data, we found no evidence that the genetic risk factors for MS also played a role in SVD as estimated by the extent of WMH.

One interpretation is that inflammatory mechanisms do not play a causal role in ischemic WMH. However, given that SNPs that associate significantly with MS largely implicate particular immune genes, an alternative possibility is that different immune pathways are relevant in each of the diseases. SNPs that associate significantly with WMH lesions principally code for extracellular matrix proteins and cell adhesion molecules. It may be that the inflammatory activation within a defined MS lesion is mediated by a different pathway than the more generalized cellular response to hypoxia and differing metalloprotease expression between ischemic and MS lesions has previously been shown.^38^ In addition, 2 traits which do not share genetic risk might nevertheless act via the same cellular pathway activated by distinct triggers. It is also possible that common biological mechanisms might have opposite associations, leading to overall null results.

Estimation of the reciprocal effect of SNPs significant for WMHs on the risk of MS added robustness, however, analysis of the effect of these SNPs was limited by the relatively small number available to factor into any polygenic risk score calculations. We did not have direct access to genotype-level MS data meaning, we were not able to derive polygenic risk scores in MS data, which would have enabled more detailed analysis of the effect of WMH-associated SNPs in MS.

Another limitation of our study is that we while we took the volume of WMHs as our measure of the extent of ischemic white matter damage the genetic data on MS looked instead at the risk of the disease rather than the extent of white matter lesions; such data are not available in the MS consortium. We also estimated WMHs in both a stroke population and a community population. However, the vast majority of WMHs in community populations are thought to have an ischemic basis, and previous studies have shown a close genetic correlation between WMH in stroke populations and community populations.^18^ One potential explanation for negative findings such as this is lack of study power. However, the sample sizes in this study were large, suggesting that if a substantial overlap exists between MS and SVD, we would have expected to identify it.

In summary, our results do not demonstrate sharing of genetic risk between MS and vascular white matter disease and so provide no evidence to support a shared cellular pathway or pathological mechanism in the development of these conditions.

## Acknowledgments

We are grateful to the International Multiple Sclerosis Genetics Consortium for the provision of summary statistics from their latest meta-analysis.

## Sources of Funding

This study was supported by a programme grant from the British Heart Foundation (RG/16/4/32218). Dr Burgess is supported by a Sir Henry Dale Fellowship jointly funded by the Wellcome Trust and the Royal Society (Grant Number 204623/Z/16/Z). H.S. Markus is funded by a National Institute for Health Research Senior Investigator Award, and his work is supported by the Cambridge University National Health Service Trust Biomedical Research Centre.

## Disclosures

None.

## Supplementary Material

## References

[1] Debette S, Markus HS (2010). The clinical importance of white matter hyperintensities on brain magnetic resonance imaging: systematic review and meta-analysis.. BMJ.

[2] Rosenberg GA (2009). Inflammation and white matter damage in vascular cognitive impairment.. Stroke.

[3] Gouw AA, Seewann A, van der Flier WM, Barkhof F, Rozemuller AM, Scheltens P (2011). Heterogeneity of small vessel disease: a systematic review of MRI and histopathology correlations.. J Neurol Neurosurg Psychiatry.

[4] Bjerke M, Zetterberg H, Edman Å, Blennow K, Wallin A, Andreasson U (2011). Cerebrospinal fluid matrix metalloproteinases and tissue inhibitor of metalloproteinases in combination with subcortical and cortical biomarkers in vascular dementia and Alzheimer’s disease.. J Alzheimers Dis.

[5] Fornage M, Chiang YA, O’Meara ES, Psaty BM, Reiner AP, Siscovick DS (2008). Biomarkers of inflammation and MRI-defined small vessel disease of the brain: the Cardiovascular Health Study.. Stroke.

[6] Wright CB, Moon Y, Paik MC, Brown TR, Rabbani L, Yoshita M (2009). Inflammatory biomarkers of vascular risk as correlates of leukoariosis.. Stroke.

[7] Shoamanesh A, Preis SR, Beiser AS, Vasan RS, Benjamin EJ, Kase CS (2015). Inflammatory biomarkers, cerebral microbleeds, and small vessel disease: Framingham Heart Study.. Neurology.

[8] Rosenberg GA, Bjerke M, Wallin A (2014). Multimodal markers of inflammation in the subcortical ischemic vascular disease type of vascular cognitive impairment.. Stroke.

[9] Hassan A, Hunt BJ, O’Sullivan M, Parmar K, Bamford JM, Briley D (2003). Markers of endothelial dysfunction in lacunar infarction and ischaemic leukoaraiosis.. Brain.

[10] Markus HS, Hunt B, Palmer K, Enzinger C, Schmidt H, Schmidt R (2005). Markers of endothelial and hemostatic activation and progression of cerebral white matter hyperintensities: longitudinal results of the Austrian Stroke Prevention Study.. Stroke.

[11] Wakita H, Tomimoto H, Akiguchi I, Kimura J (1994). Glial activation and white matter changes in the rat brain induced by chronic cerebral hypoperfusion: an immunohistochemical study.. Acta Neuropathol.

[12] Jalal FY, Yang Y, Thompson J, Lopez AC, Rosenberg GA (2012). Myelin loss associated with neuroinflammation in hypertensive rats.. Stroke.

[13] Moutsianas L, Jostins L, Beecham AH, Dilthey AT, Xifara DK, Ban M, International IBD Genetics Consortium (IIBDGC) (2015). Class II HLA interactions modulate genetic risk for multiple sclerosis.. Nat Genet.

[14] Beecham AH, Patsopoulos NA, Xifara DK, Davis MF, Kempinnen A, Cotsapas C (2013). Analysis of immune-related loci identified 48 new susceptibility variants for multiple sclerosis.. Nat Genet.

[15] Sawcer S, Hellenthal G, Pirinen M, Spencer CC, Patsopoulos NA, Moutsianas L (2011). Genetic risk and a primary role for cell-mediated immune mechanisms in multiple sclerosis.. Nature.

[16] Fowler JH, McQueen J, Holland PR, Manso Y, Marangoni M, Scott F (2018). Dimethyl fumarate improves white matter function following severe hypoperfusion: involvement of microglia/macrophages and inflammatory mediators.. J Cereb Blood Flow Metab.

[17] Traylor M, Adib-Samii P, Harold D, Dichgans M, Williams J, Lewis CM, Alzheimer’s Disease Neuroimaging Initiative; International Stroke Genetics Consortium (ISGC), UK Young Lacunar Stroke DNA resource; METASTROKE; International Genomics of Alzheimer’s Project (IGAP), Investigators (2016). Shared genetic contribution to Ischaemic Stroke and Alzheimer’s Disease.. Ann Neurol.

[18] Traylor M, Zhang CR, Adib-Samii P, Devan WJ, Parsons OE, Lanfranconi S, International Stroke Genetics Consortium (2016). Genome-wide meta-analysis of cerebral white matter hyperintensities in patients with stroke.. Neurology.

[19] Patsopoulos NA, Baranzini SE, Santaniello A, Shoostari P, Cotsapas C, Wong G (2017). The Multiple Sclerosis Genomic Map: role of peripheral immune cells and resident microglia in susceptibility (in press).. BioRxiv.

[20] Polman CH, Reingold SC, Edan G, Filippi M, Hartung HP, Kappos L (2005). Diagnostic criteria for multiple sclerosis: 2005 revisions to the “McDonald Criteria”.. Ann Neurol.

[21] Grimaud J, Lai M, Thorpe J, Adeleine P, Wang L, Barker GJ (1996). Quantification of MRI lesion load in multiple sclerosis: a comparison of three computer-assisted techniques.. Magn Reson Imaging.

[22] Griffanti L, Zamboni G, Khan A, Li L, Bonifacio G, Sundaresan V (2016). BIANCA (Brain Intensity AbNormality Classification Algorithm): a new tool for automated segmentation of white matter hyperintensities.. Neuroimage.

[23] Miller KL, Alfaro-Almagro F, Bangerter NK, Thomas DL, Yacoub E, Xu J (2016). Multimodal population brain imaging in the UK Biobank prospective epidemiological study.. Nat Neurosci.

[24] Wakana S, Caprihan A, Panzenboeck MM, Fallon JH, Perry M, Gollub RL (2007). Reproducibility of quantitative tractography methods applied to cerebral white matter.. Neuroimage.

[25] Rutten-Jacobs LCA, Tozer DJ, Duering M, Malik R, Dichgans M, Markus HS (2018). Genetic study of white matter integrity in UK biobank (N=8448) and the overlap with stroke, depression, and dementia.. Stroke.

[26] Machiela MJ, Chanock SJ (2015). LDlink: a web-based application for exploring population-specific haplotype structure and linking correlated alleles of possible functional variants.. Bioinformatics.

[27] Verhaaren BF, Debette S, Bis JC, Smith JA, Ikram MK, Adams HH (2015). Multiethnic genome-wide association study of cerebral white matter hyperintensities on MRI.. Circ Cardiovasc Genet.

[28] Traylor M, Tozer DJ, Croall ID, Lisiecka Ford DM, Olorunda AO, Boncoraglio G (2019). Novel association with PLEKHG1 in a genome-wide analysis of white matter hyperintensities in 11,226 subjects.. Neurology.

[29] Ehret GB, Munroe PB, Rice KM, Bochud M, Johnson AD, Chasman DI (2011). Genetic variants in novel pathways influence blood pressure and cardiovascular disease risk.. Nature.

[30] Burgess S, Butterworth AS, Thompson JR (2016). Beyond Mendelian randomization: how to interpret evidence of shared genetic predictors.. J Clin Epidemiol.

[31] R Core Team (2017). R: A Language and Environment for Statistical Computing.

[32] Johnson T (2013). gtx: Genetics ToolboX. R package version 0.0.8.. https://CRAN.R-project.org/package=gtx.

[33] Purcell SM, Wray NR, Stone KL, Visscher PM, O’Donovan MC, Sullivan PF (2009). Common polygenic variation contributes to risk of schizophrenia and bipolar disorder.. Nature.

[34] Bulik-Sullivan BK, Loh PR, Finucane HK, Ripke S, Yang J, Patterson N, Schizophrenia Working Group of the Psychiatric Genomics Consortium (2015). LD Score regression distinguishes confounding from polygenicity in genome-wide association studies.. Nat Genet.

[35] Lawrence AJ, Patel B, Morris RG, MacKinnon AD, Rich PM, Barrick TR (2013). Mechanisms of cognitive impairment in cerebral small vessel disease: multimodal MRI results from the St George’s cognition and neuroimaging in stroke (SCANS) study.. PLoS One.

[36] O’Dwyer L, Lamberton F, Bokde AL, Ewers M, Faluyi YO, Tanner C (2011). Multiple indices of diffusion identifies white matter damage in mild cognitive impairment and Alzheimer’s disease.. PLoS One.

[37] Smith GD, Ebrahim S (2003). ‘Mendelian randomization’: can genetic epidemiology contribute to understanding environmental determinants of disease?. Int J Epidemiol.

[38] Anthony DC, Ferguson B, Matyzak MK, Miller KM, Esiri MM, Perry VH (1997). Differential matrix metalloproteinase expression in cases of multiple sclerosis and stroke.. Neuropathol Appl Neurobiol.

