# Supplementary material for: Do Cerebral Small Vessel Disease and Multiple Sclerosis Share Common Mechanisms of White Matter Injury? A Genetic Study
Source: Stroke. 2019 Jun 21;50(8):1968–72. doi: 10.1161/STROKEAHA.118.023649 (PMC6661245; doi:10.1161/STROKEAHA.118.023649)
Supplement: Supplementary file 1 [file str-50-1968-s001.pdf]

SUPPLEMENTAL MATERIAL

Do cerebral small vessel disease and multiple sclerosis share common mechanisms of white matter injury? A genetic study.

Robin B. Brown MA[1*], Matthew Traylor PhD[1], Stephen Burgess PhD[2,3], Stephen Sawcer PhD[1], Hugh S. Markus FMedSci[1]

[1]Department of Clinical Neurosciences, University of Cambridge, Cambridge, UK

[2]MRC Biostatistics Unit, Cambridge Institute of Public Health, Cambridge, UK

[3]Cardiovascular Epidemiology Unit, Department of Public Health and Primary Care, University of Cambridge, Cambridge, UK

*Corresponding author

Email: rbb29@cam.ac.uk

Telephone: +44 01223 216509

Twitter: #CamStroke

Cover title: Do SVD and MS share pathogenesis? A genetic study.

Tables: 1

Indexing terms: *cerebral small vessel disease; multiple sclerosis; genetic association studies;*

Subject terms: *Cerebrovascular Disease/Stroke; Genetic Association Studies; Ischaemia; Inflammation; Blood-Brain Barrier*

Supplemental tables

Supplemental Table I shows the list of SNPs associated with MS in published GWAS[1,2] that were taken forward for analysis, and the summary statistics from our data used for analysis. Where a variant was identified by Sawcer et al. and confirmed by Beecham et al., we used the summary statistics published in the most recent meta-analysis.

Supplemental Table II shows the list of SNPs associated with WMH volume in published GWAS[3,4,5] that were taken forward for analysis and the summary statistics used for analysis. Where a variant was identified by Verhaaren et al. and confirmed by Traylor et al., we used the summary statistics published in the most recent meta-analysis.

Supplemental Table III gives the results of sensitivity analyses for our calculated summary statistic association scores, stratified by population, measure and exclusion of minor alleles below specific frequencies.

References

1. Beecham AH, Patsopoulos NA, Xifara DK, Davis MF, Kempinnen A, Cotsapas C et al. Analysis of immune-related loci identified 48 new susceptibility variants for multiple sclerosis. Nat Genet 2013;45:1353-1360.
2. Sawcer S, Hellenthal G, Pirinen M, Spencer CC, Patsopoulos NA, Moutsianas L et al. Genetic risk and a primary role for cell-mediated immune mechanisms in multiple sclerosis. Nature 2011; 476:214-219.
3. Verhaaran BF, Debette S, Bis JC, Smith JA, Ikram MK, Adams HH et al. Multiethnic Genome-Wide Association Study of Cerebral White Matter Hyperintensities on MRI. Circ Cardiovasc Genet 2015;8:398-409
4. Traylor M, Zhang CR, Adib-Samii P, Devan WJ, Parsons OE, Lanfranconi S et al. Genome-wide meta-analysis of cerebral white matter hyperintensities in patients with stroke. Neurology 2016;86:146-153.
5. Traylor M, Tozer DJ, Croall ID, Lisiecka Ford DM, Olorunda AO, Boncoraglio G et al. Novel Association with PLEKHG1 in a Genome-wide Analysis of White Matter Hyperintensities in 11,226 Subjects. Neurology 2019;92:e749-e757

**Supplemental table I.** SNPs associated with multiple sclerosis at genome-wide significance													
**Variant**	**Location (chromosome and base position)**	**Gene(s)**	**Reference**	**Beta coefficient of risk score (MS)**	**P value (MS)**	**Beta coefficient (WMH volume – WMH in stroke)**	**P value**	**Beta coefficient (WMH volume – UK Biobank)**	**P value**	**Beta coefficient (FA – UK Biobank)**	**P value**	**Beta coefficient (MD – UK Biobank)**	**P value**
rs1014486	chr3:159872508	IL12A	[1]	0.104	1.10e-18	0.0599	0.148	-0.00561	0.669	0.13009	0.039	-0.04768	0.439
rs10201872	chr2:230185270	SP140	[2]	0.086	1.80e-10	0.0211	0.699	0.010513	0.528	0.041284	0.606	-0.00052	0.995
rs1021156	chr8:78501108	ZC2HC1A	[1]	0.113	8.50e-17	0.0619	0.183	-0.00699	0.639	-0.05274	0.461	0.013527	0.847
rs10466829	chr12:9364013	CLECL1	[2]	0.086	1.40e-08	NA	N/A	-0.00962	0.457	-0.01723	0.782	0.018122	0.766
rs1077667	chr19:6613822	TNFSF14	[1],[2]	0.148	1.70e-24	-0.0205	0.684	-0.03356	0.034	-0.1064	0.161	0.109606	0.141
rs11052877	chr12:9410118	CD69	[1]	0.095	5.60e-13	NA	N/A	0.007516	0.576	-0.04883	0.449	0.023133	0.714
rs11154801	chr6:135268129	AHI1	[1],[2]	0.104	1.80e-20	0.0423	0.318	0.007163	0.592	0.003239	0.96	0.004219	0.946
rs1131265	chr3:119384793	TIMMDC1	[1]	0.174	1.40e-23	-0.0845	0.106	-0.01547	0.348	-0.03916	0.621	-0.05191	0.503
rs11554159	chr19:18080811	IFI30	[1]	0.14	1.90e-24	-0.0619	0.17	-0.01737	0.23	0.029225	0.675	0.012197	0.858
rs11587876	chr1:85215222	DDAH1	[1]	0.113	4.40e-09	0.0014	0.979	0.007308	0.648	0.02074	0.788	-0.01333	0.86
rs11719975	chr3:18566300		[1]	0.086	1.10e-08	-0.0507	0.279	0.008375	0.563	0.024922	0.72	-0.07214	0.289
rs12087340	chr1:85138055	BCL10	[1]	0.199	1.10e-20	-0.0461	0.534	-0.01095	0.644	-0.04098	0.72	0.004674	0.967
rs12148050	chr14:102585564	TRAF3	[1]	0.077	5.10e-13	-0.025	0.555	0.00322	0.811	0.134338	0.038	-0.08048	0.204
rs12149527	chr16:79072922	WWOX	[1]	0.077	3.30e-11	-0.0417	0.318	-0.00784	0.544	0.004893	0.937	-0.06998	0.249
rs12296430	chr12:6373620	LTBR	[1]	0.131	7.20e-14	-0.151	0.008	0.018457	0.269	0.071691	0.372	-0.0105	0.894

rs12368653	chr12:57567711	AGAP2	[2]	0.104	1.70e-09	-0.0394	0.341	0.002626	0.841	-0.07283	0.245	0.026147	0.67
rs12466022	chr2:43085671		[2]	0.095	6.20e-10	0.0131	0.776	-0.00379	0.794	-0.02367	0.735	0.050468	0.461
rs12927355	chr16:10923201	CLEC16A	[1]	0.182	6.40e-46	-0.0657	0.127	0.015733	0.255	0.029656	0.656	0.000853	0.99
rs12946510	chr17:39219977	IKZF3	[1]	0.077	2.90e-09	-0.0356	0.395	0.011782	0.36	0.027426	0.657	-0.03259	0.59
rs1359062	chr1:192500477	RGS1	[1]	0.166	4.80e-20	0.0309	0.558	0.013597	0.415	0.056031	0.485	-0.01593	0.839
rs140522	chr22:50491152	ODF3B	[2]	0.086	1.70e-08	0.0438	0.308	0.010029	0.474	0.032437	0.63	-0.02094	0.751
rs17066096	chr6:137006677	IL22RA2	[1],[2]	0.131	1.60e-23	-0.0079	0.868	0.00169	0.91	-0.02903	0.687	0.019908	0.778
rs17119	chr6:14691381		[1]	0.104	1.00e-10	0.0617	0.226	-0.01287	0.43	0.021976	0.779	-0.09017	0.24
rs17174870	chr2:111886554	MERTK	[2]	0.095	1.30e-08	-0.0477	0.317	0.004741	0.752	0.03557	0.622	-0.05442	0.441
rs17785991	chr20:49790590	SLC9A8	[1]	0.086	4.20e-08	-0.0599	0.16	-0.0091	0.508	-0.03868	0.558	-0.04946	0.444
rs1782645	chr10:79252314	ZMIZ1	[1]	0.086	2.50e-15	0.0377	0.379	0.006436	0.625	-0.00489	0.938	0.023697	0.702
rs1800693	chr12:6312329	TNFRSF1A	[1],[2]	0.131	6.70e-28	-0.1107	0.009	0.025316	0.055	0.001554	0.98	0.053393	0.39
rs1813375	chr3:27999281		[1]	0.14	1.90e-32	0.0313	0.442	-0.00685	0.597	0.054955	0.377	-0.00022	0.997
rs1843938	chr7:3053866	CARD11	[1],[2]	0.077	1.20e-10	-0.0118	0.777	0.009357	0.469	-0.03814	0.54	0.028274	0.642
rs1870071	chr19:16328687	EPS15L1	[1]	0.113	2.00e-15	0.0112	0.801	0.000805	0.954	0.014194	0.831	0.025953	0.69
rs1886700	chr16:68402003	CDH3	[1]	0.104	1.30e-08	-0.0054	0.924	0.011843	0.526	-0.1111	0.214	0.158487	0.07
rs1920296	chr3:121616614	IQCB1	[1]	0.131	6.50e-22	-0.0292	0.498	0.012624	0.346	0.008456	0.895	-0.03017	0.632
rs2050568	chr1:157516546	FCRL1	[1]	0.077	1.50e-10	-0.0166	0.689	-0.00646	0.62	-0.01304	0.835	-0.05377	0.38
rs2104286	chr10:5988280	IL2RA	[1]	0.182	2.30e-47	-0.0193	0.682	-0.00092	0.95	0.000978	0.989	-0.04497	0.513

rs212405	chr6:158901294	TAGAP	[1]	0.14	8.00e-21	0.0398	0.357	0.003304	0.806	0.020619	0.75	0.000719	0.991
rs2163226	chr2:43092906		[1]	0.095	2.10e-16	-0.0062	0.908	-0.01067	0.458	-0.00975	0.888	0.030133	0.656
rs2236262	chr14:68696738	ZFP36L1	[1]	0.077	2.50e-12	0.001	0.98	0.002566	0.844	0.076581	0.221	-0.17656	0.004
rs2248359	chr20:54160302	CYP24A1	[1],[2]	0.077	2.00e-13	-0.0602	0.149	0.006327	0.635	0.063322	0.322	-0.05726	0.36
rs2255214	chr3:121849748	CD86	[1]	0.131	1.20e-24	-0.042	0.31	0.013182	0.309	0.047044	0.45	-0.05283	0.386
rs2256814	chr20:63568094	SLC2A4RG	[1]	0.104	3.50e-09	0.0633	0.239	0.014395	0.369	0.021274	0.782	0.070607	0.348
rs2283792	chr22:21644220	MAPK1	[1],[2]	0.077	5.50e-16	-0.0442	0.275	0.008096	0.534	-0.0461	0.461	0.081934	0.181
rs2288904	chr19:10523588	SLC44A2	[1]	0.131	1.60e-11	0.0624	0.205	0.005803	0.709	0.037804	0.613	-0.02409	0.742
rs2300603	chr14:75476220	BATF	[2]	0.104	2.00e-08	-0.0768	0.095	-0.0031	0.831	-0.0153	0.827	-0.02427	0.723
rs2371108	chr3:27639513	EOMES	[1]	0.077	1.50e-15	0.0178	0.663	-0.03051	0.023	0.143048	0.026	-0.09677	0.124
rs2456449	chr8:127078717		[1]	0.095	1.80e-09	-0.0081	0.849	-0.00551	0.684	0.053178	0.413	-0.04657	0.464
rs2546890	chr5:159091760	IL12B	[2]	0.095	1.20e-11	0.0139	0.735	0.002845	0.826	0.012818	0.836	-0.06097	0.315
rs2688608	chr10:73639542	C10orf55	[1]	0.077	4.60e-08	-0.0262	0.524	0.01242	0.342	-0.0957	0.128	0.056843	0.355
rs2726518	chr4:105002488	TET2	[1]	0.086	3.90e-08	0.1134	0.006	-0.00023	0.986	0.014029	0.822	0.024077	0.693
rs3007421	chr1:6276497	PLEKHG5	[1]	0.113	4.70e-10	-0.1753	0.008	0.004428	0.818	0.001376	0.988	-0.01618	0.858
rs34383631	chr11:60971360-61068645	CD6	[1]	0.104	3.70e-23	-0.0268	0.53	-0.01654	0.216	0.004437	0.945	-0.01565	0.803
rs34536443	chr19:10280033	TYK2	[1]	0.247	1.80e-14	0.0744	0.517	0.016797	0.604	-0.15634	0.316	0.024038	0.875
rs354033	chr7:149421444	ZNF767P	[2]	0.095	4.70e-09	0.011	0.812	0.006315	0.672	0.062103	0.386	-0.01199	0.864
rs35929052	chr16:85948640	IRF8	[1]	0.131	5.90e-12	0.079	0.244	0.009729	0.631	-0.09558	0.325	0.110691	0.244

rs35967351	chr1:160710974	SLAMF7	[1]	0.086	4.40e-11	-0.092	0.034	-0.0064	0.641	-0.04364	0.509	0.064878	0.316
rs3748817	chr1:2431888	MMEL1	[1]	0.131	1.30e-26	0.0239	0.574	-0.01733	0.203	0.05914	0.366	-0.01327	0.836
rs41286801	chr1:92163769	EVI5	[1]	0.182	1.40e-26	0.1669	0.006	-0.01739	0.334	0.063049	0.465	-0.11211	0.184
rs4285028	chr3:121619717	SLC15A2	[2]	0.104	1.80e-08	-0.0115	0.807	-0.00517	0.729	-0.008	0.911	0.087294	0.214
rs4410871	chr8:127741008	PVT1,MIR1204	[1],[2]	0.113	4.30e-16	-0.0352	0.443	-0.00389	0.786	-0.01652	0.81	-0.00394	0.953
rs4665719	chr2:24464482	CENPO	[1]	0.086	3.10e-09	0.0345	0.45	-0.01775	0.234	-0.02362	0.741	0.048553	0.488
rs4679081	chr3:32856713	CCR4	[1]	0.077	2.20e-09	0.0514	0.246	-0.00058	0.966	0.035857	0.578	-0.04441	0.482
rs4772201	chr13:99392419	MIR548AN	[1]	0.113	1.30e-10	-0.0068	0.899	-0.00601	0.722	-0.11296	0.164	0.048009	0.546
rs4794058	chr17:47231738	NPEPPS	[1]	0.077	1.00e-13	0.0637	0.123	0.016217	0.209	-0.05907	0.34	0.054965	0.365
rs4796791	chr17:42156716	STAT3	[1]	0.095	3.70e-20	0.0501	0.247	-0.00981	0.468	-0.02362	0.715	-0.02236	0.724
rs4810485	chr20:45910853	CD40	[1]	0.077	7.70e-16	-0.0315	0.503	-0.01113	0.458	-0.07049	0.327	0.030036	0.67
rs4976646	chr5:177079730	RGS14	[1]	0.122	4.40e-18	0.006	0.893	0.020562	0.135	-0.07795	0.238	0.005149	0.937
rs533646	chr11:118441401	TREH	[1]	0.095	7.60e-11	-0.0355	0.42	-0.00839	0.54	0.065799	0.317	0.055276	0.39
rs55838263	chr1:200892467	C1orf106	[1]	0.113	4.00e-19	-0.0524	0.257	-0.01106	0.435	-0.04987	0.463	-0.0264	0.692
rs59772922	chr15:78696056	CTSH	[1]	0.104	1.20e-08	-0.0131	0.806	-0.00051	0.976	0.108882	0.182	-0.05608	0.483
rs60600003	chr7:37318708	ELMO1	[1]	0.148	6.00e-14	-0.1151	0.1	0.030784	0.155	-0.02228	0.831	0.08694	0.394
rs6498184	chr16:11227066	RMI2	[1]	0.14	7.40e-18	0.0044	0.935	0.007741	0.641	-0.02141	0.788	-0.02457	0.753
rs650258	chr11:60990871	CD5	[2]	0.113	2.00e-11	-0.0255	0.601	0.000929	0.944	-0.05424	0.393	0.014092	0.821
rs666930	chr1:119621680	PHGDH	[1]	0.086	6.00e-12	-0.0501	0.228	0.001852	0.885	-0.06059	0.326	0.073919	0.221

rs6677309	chr1:116487687	CD58	[1]	0.285	5.40e-42	-0.0952	0.116	-0.00036	0.985	0.024577	0.793	0.029348	0.749
rs67297943	chr6:137749522	TNFAIP3	[1]	0.113	5.50e-13	-0.007	0.894	-0.02261	0.157	-0.08374	0.276	0.12234	0.104
rs6880778	chr5:40286865		[1]	0.095	8.10e-20	-0.0073	0.861	-0.00719	0.59	0.041343	0.519	-0.05639	0.369
rs6881706	chr5:35797278	IL7R	[1]	0.113	4.30e-17	-0.0096	0.834	0.009316	0.521	-0.06658	0.34	-0.0221	0.746
rs694739	chr11:64076473	PRDX5	[1],[2]	0.077	2.00e-09	-0.0258	0.549	0.028541	0.03	0.003486	0.956	-0.00167	0.979
rs706015	chr7:26619513		[1]	0.131	1.10e-09	0.0593	0.261	-0.00904	0.587	0.070984	0.373	0.006875	0.93
rs7120737	chr11:46326774	AGBL2	[1]	0.122	1.00e-09	-0.0937	0.117	0.021317	0.241	0.032115	0.713	0.052974	0.536
rs7132277	chr12:122875604	PITPNM2	[1]	0.095	1.90e-13	0.0221	0.682	-0.00768	0.645	-0.03099	0.698	0.039312	0.615
rs71624119	chr5:56122309	ANKRD55	[1]	0.113	3.40e-13	-0.0282	0.58	0.018597	0.218	0.079727	0.271	-0.04204	0.553
rs7196953	chr16:79595363	MAF	[1]	0.077	1.00e-10	0.0231	0.601	0.007137	0.613	-0.00089	0.99	0.051049	0.441
rs7204270	chr16:29834364	MAPK3	[1]	0.086	1.60e-11	-0.0409	0.317	-0.00239	0.854	-0.18806	0.002	0.148548	0.015
rs7238078	chr18:58650125	MALT1	[2]	0.104	2.50e-09	-0.0206	0.66	0.004958	0.744	0.024316	0.739	-0.01319	0.853
rs72928038	chr6:90097116	BACH2	[1]	0.104	1.50e-15	-0.0806	0.163	0.015396	0.359	-0.19934	0.014	0.127012	0.108
rs74796499	chr14:87857711	GALC	[1]	0.278	2.40e-20	-0.0425	0.651	-0.01843	0.532	0.178689	0.209	-0.05724	0.681
rs7552544	chr1:100744097	VCAM1	[1]	0.077	1.90e-16	-0.0317	0.447	0.006868	0.602	0.013896	0.826	-0.06711	0.277
rs756699	chr5:134069014	TCF7	[1],[2]	0.113	8.80e-11	0.1304	0.026	0.010746	0.584	0.111801	0.235	-0.0812	0.378
rs7595717	chr2:68308313	PLEK	[1]	0.095	1.20e-13	-0.0088	0.849	-0.04934	0.001	0.05101	0.478	-0.03371	0.632
rs759648	chr8:128105690	MIR1208	[1]	0.086	5.00e-10	0.0553	0.226	0.015707	0.268	-0.06249	0.359	0.038622	0.563
rs7665090	chr4:102467408	MANBA	[1]	0.077	1.00e-08	0.0341	0.403	-0.01541	0.229	-0.07377	0.231	0.041315	0.493

rs771767	chr3:101953037	NFKBIZ	[2]	0.113	8.60e-09	0.0106	0.811	-0.01137	0.428	-0.12713	0.065	0.082679	0.22
rs7769192	chr6:137552553		[1]	0.077	3.30e-09	-0.0385	0.353	-0.00874	0.501	-0.02976	0.634	0.039044	0.523
rs7923837	chr10:92439580	HHEX	[1],[2]	0.104	4.30e-17	0.0386	0.361	-0.01727	0.193	0.026077	0.682	-0.05176	0.406
rs793108	chr10:30843546		[1],[2]	0.086	6.10e-12	0.016	0.701	0.007023	0.587	-0.06336	0.307	0.155135	0.011
rs802734	chr6:127633469	PTPRK	[2]	0.095	5.50e-09	0.0366	0.406	0.00823	0.56	-0.01269	0.851	0.05056	0.446
rs8042861	chr15:90268570	IQGAP1	[1]	0.077	2.20e-09	-0.0675	0.104	-0.00715	0.584	0.015367	0.806	0.001428	0.981
rs8070345	chr17:59412005	VMP1	[1]	0.131	2.20e-23	-0.0008	0.984	0.00178	0.891	-0.12344	0.047	0.101148	0.097
rs8107548	chr19:49217653	DKKL1	[1]	0.086	5.70e-15	-0.0473	0.31	-0.02187	0.14	-0.00126	0.986	0.034775	0.617
rs842639	chr2:60694033	LINC01185	[1]	0.104	2.00e-14	-0.0746	0.091	0.001665	0.903	-0.00667	0.919	-0.03358	0.601
rs917116	chr7:28080094	JAZF1	[1]	0.113	3.30e-09	-0.039	0.431	-0.00568	0.718	-0.16052	0.033	0.094781	0.199
rs941816	chr6:36376436	PXT1	[1]	0.122	3.90e-12	-0.0465	0.359	-0.01231	0.459	-0.00195	0.981	0.023729	0.762
rs9736016	chr11:118523399	CXCR5	[1]	0.095	3.00e-15	-0.0138	0.747	-0.01153	0.387	-0.05047	0.431	0.007084	0.91
rs9828629	chr3:71383457	FOXP1	[1]	0.077	1.90e-10	0.0185	0.666	-0.01779	0.185	-0.04948	0.442	0.078214	0.215
rs9967792	chr2:191050989	STAT4	[1]	0.104	3.50e-12	-0.0174	0.681	-0.00657	0.624	0.019243	0.765	-0.02312	0.713
rs9989735	chr2:230211574	SP140	[1]	0.157	4.20e-23	0.0028	0.959	0.009982	0.541	0.093424	0.234	-0.01919	0.803

**Supplemental table II.** SNPs associated with WMH volume at genome-wide significance							
**Variant**	**Location (chromosome and base position)**	**Gene(s)**	**Reference**	**Beta coefficient of risk score (WMH)**	**P value (WMH)**	**Beta coefficient of risk score (MS)**	**P value (MS)**
rs11679640	chr2:43141485	HAAO	[3]	0.051	4.4e-8	0.0197	0.585
rs275350	chr6:150120999	PLEKHG1	[5]	0.071	1.6e-8	-0.0192	0.107
rs2984613	chr1:156197380	PMF-BGLAP	[3]	0.020	2.0e-8	0.0082	0.0079
rs7214628	chr17:73882148	TRIM65	[3],[4]	0.077	2.4e-15	0.0097	0.001
rs72934505	chr2:203916487	NBEAL1	[4]	0.064	2.2e-8	-0.0066	9e-5
rs78857879	chr2:56135099	EFEMP1	[4]	0.104	1.5e-8	-0.0074	0.124
rs7894407	chr10:105176179	PDCD11	[3]	0.020	2.6e-8	0.0036	0.194
rs7909791	chr10:105613178	SH3PXD2A	[3],[4]	0.049	2.9e-9	0.001	0.328
rs941898	chr14:100599437	EVL	[4]	0.095	4.0e-8	-0.0106	0.892
rs9515201	chr13:111040798	COL4A2	[4]	0.086	6.9e-9	-0.0299	0.648
rs962888	chr17:43059071	C1QL1	[4]	0.086	1.1e-8	-0.0115	0.174

**Supplemental table III.** Sensitivity analyses for genetic associations between dependent and independent variable risk scores, stratified by measurement, testing dataset and threshold minor allele frequency. Data are relative risk scores and 95% confidence intervals.		
**Comparison (dependent vs independent variable)**	**MAF ≥ 10%**	**MAF ≥ 20%**
WMH vs MS[WMH in stroke]	1.007 (0.927 – 1.094)	1.019 (0.927 – 1.121)
WMH vs MS[UK Biobank]	1.020 (0.932 – 1.117)	1.019 (0.926 – 1.121)
FA vs MS[UK Biobank]	1.008 (0.891 – 1.141)	0.975 (0.846 – 1.123)
MD vs MS[UK Biobank]	0.971 (0.860 – 1.096)	0.990 (0.862 – 1.137)
MS vs WMH[IMSGC]	0.887 (0.747 – 1.053)	0.957 (0.780 – 1.174)
