# Supplementary material for: Do Cerebral Small Vessel Disease and Multiple Sclerosis Share Common Mechanisms of White Matter Injury? A Genetic Study
Source: Stroke. 2019 Jun 21;50(8):1968–72. doi: 10.1161/STROKEAHA.118.023649 (PMC6661245; doi:10.1161/STROKEAHA.118.023649)
Supplement: Supplementary file 2 [file str-50-1968-s002.pdf]

Change of Authorship Form

(Must be completed and signed by ALL authors)

Please check all that apply

✓ New author(s) have been added (in addition to this form, all new authors must complete the copyright transfer agreement and conflict of interest disclosure.

_____Change in order of authorship.

_____An author wishes to remove his/her name. An author's name may only be removed his/her own request and a letter signed by the author should accompany this form

Manuscript Number 023649R3

Manuscript Title Do cerebral small vessel disease and multiple sclerosis share common mechanisms of white matter injury? A genetic study.

Former Authorship

Please list ALL AUTHORS in the same order as the original submission. For more than 12, use an extra sheet.

Print Name		Print Name	
Name (1)	ROBIN B BROWN	Name (7)	
Name (2)	MATTHEW TRAYLOR	Name (8)	
Name (3)	STEPHEN SAWCER	Name (9)	
Name (4)	HUGH S MARKUS	Name (10)	
Name (5)		Name (11)	
Name (6)		Name (12)	

New Authorship

All authors must sign below agreeing to the changes in authorship. The authorship order must reflect the authorship order of the manuscript.

Name		Signature	Date
Name (1)	ROBIN B BROWN	[signature]	18/04/19
Name (2)	MATTHEW TRAYLOR	[signature]	24/04/19
Name (3)	STEPHEN BURGESS	[signature]	25/4/2019
Name (4)	STEPHEN SAWCER	[signature]	18/4/19
Name (5)	HUGH S MARKUS	[signature]	24/4/19
Name (6)			
Name (7)			
Name (8)			
Name (9)			
Name (10)			
Name (11)			
Name (12)			

Please scan and email to stroke@strokeahajournal.org.

Scanned by CamScanner
